# The Relation of Voluntary Hospitals to the State

**Published:** 1919-05-24

**Authors:** 


### THE BRITISH HOSPITALS ASSOCIATION.

### The Relation of Voluntary Hospitals to the State.

The annual meeting of the British Hospitals Association was held on Friday, May 16, at the London Hospital, Whitechapel, the Rt. Hon. Viscount Knutsford, chairman of the Hospital, presiding.

There were present :—

H. Wade Deacon, Liverpool Royal Infirmary; Sir Arthur Stanley, G.B.E., St. Thomas's Hospital; Rev. G. B. Cronshaw, Radcliffe Infirmary, Oxford; E. W. Morris, London Hospital; C. S. Risbee, General Hospital, Northampton; P. Michelli, Seamen's Hospital; Frank G. Hazell, Manchester Royal Infirmary; P. B. Burgoyne, Royal National Hospital for Consumptives, Ventnor; George Cunningham, Middlesex Hospital; Francis Penty, York County Hospital; W. H. Harper, Wolverhampton General Hospital; Walter Banks, Derbyshire Royal Infirmary; G. H. Smith, Derbyshire Royal Infirmary; A. M. Daniel, Scarborough General Hospital; George Davidson, Aberdeen Royal Infirmary; Arthur Moore, County Hospital, Lincoln; Lord C. Courtney, Major, R.A.M.C., British Medical Association; P. J. M. Rogers, Poplar Hospital; L. Blackett, Hospital of St. John and St. Elizabeth; W. T. Patrick, Royal Surrey County Hospital; Harry A. Bone, Miller Hospital; R. J. Coles, King's College Hospital; Hy. J. Eason, London Lock Hospital; J. Courtney Buchanan, Metropolitan Hospital; J. Maddock, Wellington Hospital; C. R. W. Offen, Leamington; J. M. Churchfield, St. George's Hospital; C. E. L. Lyle, Queen Mary's Hospital; J. H. A Marchand, Victoria Cottage Hospital; Major T. Basil Rhodes; R. Ball Dodson, Royal Essex County Hospital; J. W. Barnes, Royal Infirmary, Sheffield; W. W. Parkinson, London Temperance Hospital; W. A. Tait, Royal Infirmary, Edinburgh; E. C. Smith, Queen's Hospital, Birmingham; W. G. Marten, East Ham; W. E. Budgett, Bristol Royal Infirmary; R. E. V. Bax, Seamen's Hospital Society; Capt. Darmady and James McKay, Great Ormond Street Children's Hospital; F. W. Drewett, Tottenham Hospital; H. H. Jennings, Chelsea Hospital; Sir A. K. Butterworth, Victoria Park Hospital; H. Wigg, Hampstead Hospital; R. A. Owthwaite and C. W. Thies, Hon. Treasurers.

**Viscount Knutsford** humorously introduced Sir Napier Burnett to the meeting. He said when he went to the War Office to see Sir Napier, he found he knew more about hospitals than his lordship did himself. One of the items referred to was cheese; Sir Napier told him that in some hospitals, besides using it as cheese, they melted it down and called it butter, they froze it and called it soap, they employed it for stopping bleeding, and so on. Seriously, he did not know any man who had more conscientiously studied hospitals, and the information he had given to the London and other hospitals had been of immense value to them.

### Sir E. Napier Burnett's Address.

**Sir E. Napier Burnett, K.B.E., M.D.,** in opening the discussion on "The Relation of Voluntary Hospitals to the State," said : There are strong temptations for one to cover a wide field, but as I am desirous, with your Council, that some practical result may be attained by this meeting, I have exercised the power of restraint in confining my observations to a few salient points. I suggest, at the outset, that two points may be borne in mind throughout the discussion, namely :—

(1) That we are considering the hospital situation as a whole throughout the country, not merely those hospitals which are situate in this vast Metropolis, which, doubtless, have their own peculiar advantages and problems. (2) Let us take the broad outlook towards what is in the best interests of the nation, rather than the point of view of any one individual hospital.

I realise that this audience is composed, in the main, of those who have been bred and nurtured in what is known as "the voluntary principle," and I am confident that when the full story comes to be recorded of what the voluntary hospitals have accomplished, it will be demonstrated that your beliefs have been well founded. My object therefore is not the foolish one of seeking to condemn what has been attained under voluntaryism, but, rather, to point out that some alteration in policy is required in order to harmonise the hospital system with the circumstances of to-day, which are so vastly different from those pertaining to the times when the voluntary hospitals came into being. In those early days, the so-called "sick and lame poor" constituted the hospital *clientèle*, but the discoveries of modern science applied to the treatment of disease have brought increasing numbers of people from the various grades of society to your doors in search of the latest and best forms of medical and surgical treatment. The voluntary hospitals, to the best of their ability, have responded to and are meeting this new and ever-increasing demand for treatment, with the inevitable result that the annual financial expenditure is now much in excess of the income derived from the philanthropic public. When the income of an individual (and so also of an institution) fails to meet the expenditure, then there is one doom lying ahead, namely, bankruptcy—a condition looming on the horizon for many hospitals during this present year. During the past four years, the income derived from the State for the treatment of military patients occupying beds in your hospitals, which otherwise would have been occupied by non-paying patients, as they are called, has been of considerable assistance to many hospitals in tiding them over an anxious period. The war service rendered to the State by the voluntary hospitals was a magnificent piece of work highly appreciated by the military authorities, and although the remuneration granted for such service during a time of national emergency may not have been such as some of you desired, yet its cessation would appear to be causing anxiety to certain hospital committees.

### The Attitude of the Association.

**Let me ask the question : What is the policy of this Association towards the solution of the threatening financial crisis? I suggest that there are two methods open for consideration, viz., either (1) expenditure must be curtailed, or (2) income must be increased. Let us for a moment examine these alternatives.**

### (1) Expenditure.

The curtailing of expenditure, although it may excite less interest than the increasing of income, is, nevertheless, an aspect of considerable importance in hospital administration. In the present argument I am tempted to ask whether economy, in the fullest meaning of the word, *i.e.*, everything being put to its best use—which is my definition of economy—is practised in all hospitals.

Average Stay of Patients. By Motor Ambulance to Country Convalescent Camps.

I will just refer to one point in this connection which I suggest is worthy of some consideration. In a general

**The British Hospitals Association** (*continued*).

hospital in any of our large cities the average length of patients' stay is usually between three and four weeks, and the annual cost per occupied bed will range from £120 upwards. Are these hospital beds being put to their maximum use? When the surgeon goes round his wards he recognises many patients who have had their operative treatment a week or ten days previously, and who are now lying convalescing. I suggest that after, say, ten to fourteen days such patients might be transferred by motor ambulance to convalescent homes in the country where the rate of convalescence would be accelerated by from 20 per cent. to 30 per cent. as compared with lying in a ward in the centre of some populous city amidst its noisy thoroughfares; and annual cost per occupied bed in such homes is certainly one-third less than in the general hospital. By such an arrangement the urgently needed beds in the central hospital might be put to a fuller use.

CLOSING HOSPITAL BEDS.

Prior to the war some hospitals made no effort to conceal the fact that they had been obliged to shut down one or more wards so as to lessen expenditure; but surely such a policy can have no advocate in these days, when the health needs of the nation stand revealed in all their magnitude, and when the health-conscience of the people has been quickened anew through some of the lessons of the war. "He that withholdeth, impoverisheth himself" is still sound philosophy. No hospital worthy of the name will seek to curtail expenditure by offering a limited service, or will in any way fail to make the fullest possible use of its available accommodation. On the contrary, I predict that, in the near future, the country will demand that hospital facilities be made available for a largely increased number of people. Further, the National Insurance Act may be soon so amended as to provide institutional treatment for the 13,000,000 insured people. From these observations, then, one comes to the conclusion that, contrary to there being any likelihood of decrease, the prospects are all indicative of an increased expenditure for hospital service.

**(2) Increased Hospital Income.**

Let us now look at the other alternative, namely, an increased hospital income. Hospitals have always been crying "poverty"; reforms, extensions, and improvements have been delayed by lack of funds. The efforts to raise money have been successful, or the reverse, just as there happens to be in the particular district one man skilled in the arts of the beggar to touch the hearts of the rich; hospital appeals occasionally have merged on the bounds of decency, and human suffering has been exploited by letter, by the Press, and pictorially, in order to get money. In short, hospitals are supported by charity, and it is no longer a charity which the people want. The fighting of disease is a national matter, and the workers have the right to all the protection that the State can supply. This protection requires more money than private enterprise can raise; moreover, the duty of giving is recognised unequally, some give generously, others not at all. Large employers of labour and insurance companies derive very great benefits in having their employees and clients treated at the voluntary civil hospitals, benefits received out of all proportion to the subscriptions given. With high taxation and increase of local rates, voluntary hospital subscribers are likely to diminish in number. This diminished income, *plus* the higher expenditure in nurses' salaries and general wages, tends to the embarrassment of hospital governors.

RELATION OF VOLUNTARY HOSPITALS TO THE STATE.

So much for the present hospital situation. May we now look more closely at the relationship of the voluntary hospitals to the State? I have encountered those in voluntary hospitals who hold the extreme view that to have any direct dealing with the State is anathema. I would plead to-day with any such for a recognition of the doctrine that the interests of the State must take precedence of any voluntary principle. (Hear, hear.) There are voluntary civil hospitals in the London area, as elsewhere, at present in receipt of subsidies from the State for the following services, viz., the treatment of tuberculosis, child welfare, venereal disease, pensioners, military patients, and I suggest that the treatment of these cases is not inferior because paid for by State-money rather than by voluntary subscription, and I also suggest that the hospitals in receipt of these State subsidies are no worse administered on account of such financial assistance. Let me here propound another query: Why does the State make a selection of a few diseases to be paid for? Disease is the enemy of the State, it weakens her soldiers, strikes down her workers, and decimates her future citizens. The State recognises this dimly and partially, for she has her fever hospitals, asylums, and venereal clinics. It is owing to her lack of imagination that she takes steps only when she is forced. Fevers frighten her—dire experience has taught salutary lessons; venereal disease haunts her whenever she pauses to think; she does not let her lunatics wander at large, a danger to themselves and to the lives of her peaceful citizens. The care of those who suffer from smallpox, or who are insane, or those who suffer from venereal disease, she dare not leave to private enterprise, yet she allows her workers to linger on in their dwellings unprovided for, because their disease, although it may be no less deadly to herself, does not make itself evident. Such has been the state of matters in the past, largely because the supervision of disease and all matters pertaining to health have been the business of no single State Department; no one has been wholly responsible, but now, with the establishment of a Health Ministry, all health matters will be co-ordinated under one responsible authority. Under such a Ministry I suggest that the voluntary hospitals will have an important part to play, but it must take the form of some co-ordinated scheme to replace the present unorganised and disjointed arrangement whereby each hospital is a law unto itself, offering charitable treatment how and when it pleases. If the State asks the civil hospitals to do certain work, and offers remuneration for such work, what objection can be raised by the hospitals to the State exercising a reasonable degree of control in the form of judicious inspection, with the object of ascertaining that adequate services are rendered for public moneys?

A GLORIOUS CHAPTER FOR CIVIL HOSPITALS.

The civil hospitals have a great opportunity of adding a still more glorious chapter to their history than anything they have accomplished in the past, by casting aside all prejudice, and with an open mind responding to the call of the nation for a bigger and more extended hospital service. Just as the British military hospital system throughout the war became the envy of other nations, so let me appeal to this Association to concentrate its efforts on the establishment of a co-ordinated British civil hospital system worthy of the Empire, and second to none throughout the world. (Applause.)

**The Discussion.**

**Viscount Knutsford** said that as far as this hospital (the London) was concerned, they had no objection to accepting

**The British Hospitals Association** *(continued)*.

money from the State—as much as the State chose to pay them; and it was quite reasonable that, in return for this, there should be inspection. Hospitals had already been receiving money for various purposes—for venereal disease work, for looking after children, for tuberculous cases, and so on; and these phases of medical work had helped towards the education of students, and the inspection which had followed had always been helpful. What the voluntary hospitals were afraid of, however, was not inspection, but control; what had been seen abroad concerning State control of hospitals made them anxious on this head. Sir Arthur Stanley was present, and he was a standing example of bringing a man's heart-beat into a Government Department. Technically, Sir Arthur's Red Cross work had no business to exist; it was the work of the Army, but armies had no heart, and the Red Cross Society came in and gave the human sympathy and heart for the work. Their fear was that if hospitals became an affair of the State, this last-named attribute would be lacking. To deal, under the present system, with the whole of the nation's illness was impossible. In the future he thought the hospitals would supply just what the Government would not give them under the Insurance Act. He hoped the Minister of Health would not be satisfied with the low standard of healing which obtained in the case of the panel doctor; he could not call in the aurist, the orthopædist, or any specialist to a case. He considered that the future of the voluntary hospitals would be that they would be called in as specialists to help the Minister of Health in dealing with the ordinary sickness of the nation. Members of the Association had probably changed their views a good deal since first they met Sir Napier Burnett, because their eyes had been opened to their failure to reach and deal with the vast quantity of illness in the country. He did not know that it would be much cheaper to move convalescing patients by motor ambulance to the country than to allow them to get well in the hospital where they had their operation or their acute illness. The average stay of patients at the London Hospital was twenty days. And to show that the cost of convalescence in the country was less than in a large town did not prove that such patients cost too much while they remained in town, because the earlier part of their stay was always by far the most expensive part.

**Mr. Hazell** (Royal Infirmary, Manchester) pointed out that by discharging patients to convalescent homes as soon as they were fit to be moved, the work of the wards would always be carried on at high pressure. Unless there were a number of sub-acute and convalescing patients in the wards the tension would prove too great for the nurses and other members of the staff. The same was found to apply to mental hospitals.

**Rev. G. B. Cronshaw** (Radcliffe Infirmary, Oxford) alluded to the importance of getting smaller outlying hospitals co-ordinated with the larger town hospitals, which were fully equipped with modern appliances. This would also result in an improved standard of nursing. And the smaller hospitals could be used for patients convalescing from operations performed in the larger institutions. He lost no opportunity of advocating this, and the need was all the greater in view of the expected great increase in hospital work and the building difficulties in the way of constructing new wards. He was convinced that Sir Napier Burnett's paper was on the right lines.

### Sir Arthur Stanley's Views.

**Sir Arthur Stanley, G.B.E.,** said the best proof of the way in which Sir Napier did the arduous work set him by the War Office was the fact that he had more than secured the confidence of those who had had to deal with him while he was so engaged. He could himself speak feelingly on that matter, as he was nominally responsible for 1,500 hospitals during the war, and he in that way came a good deal under Sir Napier's purview. Whenever he found that gentleman had been down to see a particular hospital, he knew there was good reason for the visit, and accordingly he looked into it without delay. If the control likely to be given to hospitals by the Government was typified by that exercised by Sir Napier, they would welcome it, to the accompaniment of any amount of money which the Government liked to give.

But there was also something to be said on the other side of the voluntary system. There was something apart from the actual value received by the patient, namely, the value received by the giver. It would be sad to see the disappearance of the voluntary principle. The Red Cross Society was the only channel through which the generosity of the public reached the sick and wounded soldier; it was the instrument through which the public expressed its feeling that everything which could be done for the wounded soldier should be done. That had a value quite distinct from that of the relief of suffering; it enabled the men who were fighting to know that this feeling on the part of the public was behind them. The grouping system referred to by Mr. Cronshaw had interested him very much; he had paid a good deal of attention to it. A very good system of auxiliary hospitals became established during the war. The military hospitals with the highest medical and surgical skill and equipment dealt with the first-line cases, and as the patients became better they were passed on to the Red Cross hospitals, which, thereby, in many instances became convalescent homes. He would like to see such a system gradually adopted in ordinary hospitals. He knew a case at the present time where there was a question of erecting a special department for a big hospital when already a special hospital for that very treatment existed only a few hundred yards away.

He agreed with all that had been said about co-ordination of work, as to the necessity of State subsidy, and about reasonable control, though, in the latter connection, he wished someone would define the word "reasonable." (Laughter.) He hoped the standard would be that set by Sir Napier Burnett himself.

### Voluntary Hospitals' Day Over?

**Mr. Wade Deacon** (Royal Infirmary, Liverpool) expressed, on his own behalf and that of the Council, their thanks to Sir Napier Burnett for his paper. There was still great waste in the ordinary general hospital. The great point was to get cases through the beds in fully equipped hospitals as quickly as possible. He had no fear as to the inspection which would accompany Government contributions; there was already a good deal of inspection, and nobody minded it, especially if there was proper management. The difficulty in his mind was as to the transition period from voluntary to subsidised. In Liverpool in 1916 the receipts were, for the first time in their history, less than the expenditure. Probably if the figures were got out for other towns a similar result would be seen. That sort of thing, obviously, could not go on indefinitely. Hospitals were jogging along, and some were using capital in order to do so, but a change was evident, and there was a laudable determination to avoid closing beds. And he had wondered how long people would go on giving towards hospitals when Government subsidies became established. That, the future alone could settle. In some of the Colonies where large sums for hospitals

**The British Hospitals Association** (*continued*).

were received from the Government, voluntary subscriptions were also continued. He felt that the day for voluntary hospitals pure and simple was now over; they were unable, under that system, to meet the requirements of the present day.

A member thought it would usually be found that when it was proposed to remove a patient to a convalescent home he insisted on going home. [Not when he knew that a journey home through the country convalescent camp meant health completely restored, fit for work.—ED., THE HOSPITAL.]

**Mr. P. B. Burgoyne** (Ventnor), representing a country hospital of 177 beds, opposed the principle of State subsidy, as it would not be able to enter into the peculiar local needs of such hospitals. His own institution collected and spent £20,000 a year, and did very well, and his committee did not look kindly on the prospect of State control.

### Matters that must be Controlled.

**Sir Napier Burnett,** answering the Chairman, said he only mentioned money as a secondary matter when referring to transfer to convalescent homes. The great point was, it would make better use of the hospital beds. He had not overlooked the point mentioned by Mr. Hazell; but the expert men and women in a hospital should be paid, and they were not being put to their best use when they were walking past convalescing patients. Shorter hours for nurses were to be anticipated. There were certain things that could be left to voluntaryism, and certain things that could not. For instance, one could not allow everybody to set up his own sanitary and lighting system, for it would mean chaos. There were certain things that had to come under control—though he hesitated to use the word. Nevertheless, any hospital could contract out of the arrangement, just as in matters of education. He would like to see the country supply the irreducible minimum of medical and surgical hospital accommodation. He did not think the State would compel a country district to come under Government control if it chose to carry on, but when they asked the Government to give help, then that help would be willingly given.

**Mr. A. M. Daniel** (Scarborough General Hospital) asked whether Sir Napier Burnett had formed any estimate of the additional number of beds that would, in his opinion, be requisite for medical and surgical cases if the whole of the insured cases were adequately treated institutionally. Until those concerned with voluntary hospitals knew how far short they fell of the requisite supply of beds, they had no really safe ground to go upon for bargaining or refusing a subsidy.

**Sir Napier Burnett** replied that a statement on the point would be made when the subject was dealt with as an amending Act.

**Mr. C. R. W. Offen** (Leamington) said they were at present in correspondence with the local town authority with regard to maternity and child welfare conditions, and were thinking of the difficulties that might arise when they had the various authorities each demanding some measure of control. He quite agreed with Sir Napier that hospitals were in a period of transition, but he hoped the policy of the Association would be that, supposing the control had to be of a representative character, it should be of a broad and enlightened type and fair with regard to the question of voluntary hospitals. Otherwise Bumbledom would again appear, and bring with it all its bad and evil ways. He hoped that the progressive principles laid down by Sir Napier Burnett would be very generously considered by every member of the British Hospitals Association, even by those who took the very old-fashioned view on the voluntary system, for they would get something better if they would only consider the greatest happiness of the greatest number.

**Sir Napier Burnett** said he had been asking for co-ordination from the hospitals, nevertheless he recognised that co-ordination among the authorities was equally necessary. So far as local authorities were concerned, he was on ground he could not follow—when they came to the rating authority, then he left it to the House of Commons!

**Mr. Wade Deacon,** in moving a vote of thanks to Sir Napier Burnett, said that in the excellent paper he had read to them he had produced the required effect—namely, providing food for thought. Moreover, he had raised a very broad question in a broad spirit. The vote of thanks having been heartily accorded,

**Sir Napier Burnett** said it had been a great pleasure to him to attend the meeting. He had been asked how he dared to enter the Holy of Holies of voluntaryism, to which he had replied, "Daniel survived; I am going to try." (Laughter.)

THE BRITISH HOSPITALS ASSOCIATION'S OFFICERS.

On the proposition of Mr. PENTY (York), seconded by Mr. WIGG, the President, Vice-Presidents, Hon. Secretaries, and Hon. Treasurer were unanimously re-elected.

THE RESIGNATION OF MR. CONRAD THIES.

Mr. WADE DEACON (Chairman of Council) expressed his and the Association's regret that the services of Mr. Conrad Thies, Hon. Secretary, were to cease in that capacity. Mr. Thies had not been favoured with good health for some time, and he felt he could no longer manage the Association's work as well as his own. He concluded by proposing a cordial vote of thanks to Mr. Thies for his able and long-continued work on the Association's behalf, and at the same time expressing the hope that he would have many years of health and happiness. This was passed.

Mr. THIES, in acknowledging the vote, said his chief consolation was that his colleague, who had been associated with him for many years (Mr. Buchanan), would continue the work. He (Mr. Thies) still felt a keen interest in the Association, and he would keep up his connection with it. The Association had been of the greatest value to hospitals; to-day it represented more than 300. Had there not been such a body to speak on behalf of hospitals as a whole when making representations to the authorities, he was sure voluntary hospitals would have suffered more than they have done; individual institutions would have no chance whatever. He had made a careful analysis of the 700 hospitals in the country, and found a large proportion of them were very small institutions, either serving rural areas or acting as auxiliary and supplementary hospitals to the city or town institutions. Therefore the membership of 429 could be claimed to represent practically all the hospitals of any weight. Some of the larger ones were represented by four or more members. The fact that men were willing to come to London to discuss hospital matters was a proof that the Association was holding together. He was extremely obliged for the vote which had been passed.

A vote of thanks to Lord Knutsford and the London Hospital authorities concluded the proceedings.

